# Unveiling genome plasticity as a mechanism of non-antifungal-induced antifungal resistance in *Cryptococcus neoformans*

**DOI:** 10.3389/fmicb.2024.1470454

**Published:** 2024-11-05

**Authors:** Lijun Zheng, Yi Xu, Liangsheng Guo

**Affiliations:** ^1^Department of Ultrasound Medicine, The Second Affiliated Hospital of Soochow University, Suzhou, China; ^2^Department of Pharmacy, The 960th Hospital of PLA, Jinan, China; ^3^Department of Obstetrics and Gynecology, The Second Affiliated Hospital of Soochow University, Suzhou, China

**Keywords:** *Cryptococcus neoformans*, tunicamycin, aneuploidy, drug resistance, fluconazole

## Abstract

*Cryptococcus neoformans*, a critical priority pathogen designated by the World Health Organization, poses significant therapeutic challenges due to the limited availability of treatment options. The emergence of antifungal resistance, coupled with cross-resistance, further hampers treatment efficacy. Aneuploidy, known for its ability to induce diverse traits, including antifungal resistance, remains poorly understood in *C. neoformans*. We investigated the impact of tunicamycin, a well-established ER stress inducer, on aneuploidy formation in *C. neoformans*. Our findings show that both mild and severe ER stress induced by tunicamycin lead to the formation of aneuploid strains in *C. neoformans*. These aneuploid strains exhibit diverse karyotypes, with some conferring resistance or cross-resistance to antifungal drugs fluconazole and 5-flucytosine. Furthermore, these aneuploid strains display instability, spontaneously losing extra chromosomes in the absence of stress. Transcriptome analysis reveals the simultaneous upregulation of multiple drug resistance-associated genes in aneuploid strains. Our study reveals the genome plasticity of *C. neoformans* as a major mechanism contributing to non-antifungal-induced antifungal resistance.

## Introduction

*Cryptococcus* species are significant contributors to opportunistic fungal infections in individuals infected with HIV worldwide. The incidence and mortality rates associated with these infections are particularly high in sub-Saharan Africa (ref). *C. neoformans* and *C. gattii* are the primary causative agents of cryptococcosis, which typically manifests as meningitis and pneumonia. The global annual estimated death toll from cryptococcal meningitis is approximately 181,100, with the majority of cases occurring in sub-Saharan Africa (Rajasingham et al., 2017). In 2022, the World Health Organization (WHO) released its inaugural list of priority fungal pathogens, categorizing *Cryptococcus neoformans*, *Candida auris*, *Aspergillus fumigatus*, and *Candida albicans* as “critical priority” pathogens (WHO, 2022).

Currently, treatment options for cryptococcosis are limited to three classes of antifungals: azoles, polyenes, and 5-flucytosine (5FC). Unfortunately, there has been a steady increase in antifungal resistance among clinical isolates of *C. neoformans*. For instance, studies of clinical isolates of *C. neoformans* collected from 134 sites across 40 countries indicated that resistance to fluconazole (FLC) increased from 7.3% between 1997 and 2000 to 11.7% between 2005 and 2007 (Pfaller et al., 2010). Today, FLC resistance is relatively common, particularly in relapse cases of cryptococcal meningitis (Bongomin et al., 2018).

Antifungal resistance often arises from genetic mutations that alter drug targets or increase drug efflux (Fisher et al., 2022). Notably, while point mutations in the *ERG11* gene can induce FLC resistance, the primary mechanism of FLC resistance observed *in vitro* and *in vivo* involves increased *ERG11* copy numbers and overexpression, facilitated by the formation of extra copies of Chromosome 1 (Sionov et al., 2010; Zafar et al., 2019; Yang et al., 2021a). Similarly, in over 50% of clinical fluconazole-resistant isolates of *C. albicans*, amplification of the left arm of Chromosome 5, which houses *ERG11* and *TAC1*, has been detected. *TAC1* encodes a transcription factor that regulates efflux pump genes (Selmecki et al., 2006; Selmecki et al., 2008). Thus, aneuploidy, which involves changes in gene copy numbers, is a common strategy used by human pathogens to rapidly adapt to stress (Tsai and Nelliat, 2019). Importantly, aneuploidy is considered as a significant mutation that can drive phenotypic changes by simultaneously altering the copy number and expression of hundreds of genes. This alteration can lead to the emergence of complex traits as a result of a single mutational event (Pavelka et al., 2010; Chen et al., 2012). Recent studies have highlighted the role of aneuploidy in mediating rapid adaptation to antifungal drugs and, in some cases, cross-adaptation to unrelated drugs in species such as *C. albicans* (Yang et al., 2019; Yang et al., 2021b), *C. parapsilosis* (Yang et al., 2021c; Sun et al., 2023a), and *C. neoformans* (Yang et al., 2021a).

The endoplasmic reticulum (ER) is a vital organelle in eukaryotic cells, responsible for protein folding and secretion. When the ER becomes overwhelmed with unfolded proteins, ER stress occurs, leading to a range of cellular responses (Luoma, 2013). Tunicamycin (TUN), a commonly used chemical, induces ER stress by inhibiting protein glycosylation, causing the accumulation of unfolded proteins (Wu et al., 2018). Recent studies have demonstrated that aneuploidy plays a crucial role in adapting to TUN-induced ER stress in yeast species. For example, in *Saccharomyces cerevisiae* and *C. albicans*, exposure to TUN primarily results in the formation of disomy of Chromosome II and trisomy of Chromosome 2, respectively. These aneuploidies are associated with specific genes that confer TUN resistance, including *ALG7*, *PRE7*, and *YBR085C-A* in *S. cerevisiae*, and *ALG7*, *RTA2*, and *RTA3* in *C. albicans* (Beaupere et al., 2018; Yang et al., 2021b). Furthermore, TUN-induced trisomy of Chromosome 2 in *C. albicans* has been shown to confer cross-adaptation to caspofungin and hydroxyurea (Yang et al., 2021b). In contrast, the mechanisms by which *C. neoformans* adapts to ER stress remain poorly understood, including whether this adaptation is accompanied by the acquisition of new traits, such as antifungal resistance.

Reference strains are a crucial component of laboratory research, providing a standardized genotype that facilitates the comparison of scientific observations within a specific species across the research community. *C. neoformans* strain H99 (ATCC 208821) was originally isolated on February 14 1978 by Dr. John Perfect at Duke University Medical Center from a 28-year-old male with Hodgkin’s disease (Janbon et al., 2014), and has since established itself as the most commonly utilized reference strain for *C. neoformans* worldwide. While several distinct lineages of H99 have arisen, displaying diverse levels of virulence as a result of storage, passage, and subculturing across various laboratories, thorough sequencing analyses of these strains have uncovered a limited number of unique mutations among them. Overall, the strains exhibit significant similarity in both growth characteristics and antifungal susceptibility (Fernandes et al., 2022). However, although H99 serves as an excellent model for grasping the fundamental biology of *C. neoformans*, conducting comparative analyses of clinical isolates will significantly enhance our understanding (Jackson et al., 2023).

In this study, we exposed the *C. neoformans* laboratory strain H99 to both mild and severe ER stresses induced by TUN to investigate the effects on antifungal resistance. Our findings showed that TUN primarily induced aneuploidy in *C. neoformans*, resulting in diverse karyotypes with recurring amplifications of Chromosomes 4 and 6, either individually or in combination with amplifications of other chromosomes. Notably, specific aneuploid strains exhibited resistance to FLC or 5FC, and some displayed cross-resistance to both drugs. We also observed that these aneuploid strains were unstable, with spontaneous loss of extra chromosomes and reversion to euploidy when grown in the absence of stress. Importantly, exposure to TUN also triggered cross-resistance to FLC in one clinical isolate of *C. neoformans*. Furthermore, our results indicate that cross-adaptation to ER stress and FLC in aneuploid strains was attributed to the simultaneous upregulation of multiple genes across the genome. This study highlights the role of ER stress in inducing aneuploidy formation and underscores the potential of non-antifungal-induced genome plasticity in driving antifungal resistance in *C. neoformans*.

## Materials and methods

### Strains and growth conditions

The *C. neoformans* laboratory strain H99 served as the wild-type strain in this study. Stock cultures were preserved in a glycerol solution and stored at a temperature of −80°C. Cells were routinely cultured in a nutrient-rich medium called Yeast extract-Peptone-Dextrose (YPD) at a temperature of 30°C in a shaking incubator set to a moderate speed of 150–200 rpm. To solidify the medium, agar was added at a concentration of 2% (w/v). The drugs used in this study were dissolved in a solvent called dimethyl sulfoxide (DMSO) and stored at a temperature of −20°C.

### Growth curves of H99 in the presence of tunicamycin

H99 was thawed from the −80°C freezer and streaked onto YPD-agar plates, which were then incubated at 30°C for 72 h. After incubation, cells were resuspended in YPD broth and their densities were adjusted to 2.5 × 10^3^ cells/mL in YPD broth with or without TUN in a 96-well plate. The TUN concentrations were 0.03–0.125 μg/mL. The plate was then incubated at 30°C, and the optical density at 595 nm (OD_595_) was monitored using a Tecan plate reader (Infinite F200 PRO, Tecan, Switzerland) at 15-min intervals for 72 h. The data are presented as the mean ± standard deviation (SD) of three biological replicates.

### Spot assay

Cells were re-suspended in distilled water and adjusted to a concentration of 1 × 10^7^ cells/mL. Following this, 3 μL of 10-fold serial dilutions were spotted onto YPD agar plates with or without the presence of drugs. The petri dish plates were then incubated at 30°C and photographed after 72 h.

### Broth microdilution assay

The broth microdilution method was performed in accordance with the Clinical and Laboratory Standards Institute (CLSI) M27-2017 guidelines (CLSI, 2017) with minor modifications.

Stock solutions of the drugs (TUN and FLC in YPD broth; 5FC in SD broth) were prepared and subjected to a twofold dilution process. A volume of 0.1 mL of each drug at varying concentrations was sequentially dispensed into U-shaped wells of 96-well microdilution plates (Thermo Fisher Scientific), resulting in final drug concentrations ranging from 0.125 to 128 μg/mL (FLC and 5FC) or 0.03–32 μg/mL (TUN).

To prepare the inoculum of test strains, 3–5 colonies were suspended in sterile saline and standardized to a turbidity of 0.5 McFarland using a spectrophotometer (NanoPhotometer® NP80, Implen, Germany). The inoculum was then diluted with either YPD broth (for TUN and FLC) or SD broth (for 5FC). Following this, 0.1 mL of the yeast suspension was added to each well of the 96-well microdilution plates, yielding a final inoculum concentration between 0.5 × 10^3^ and 2.5 × 10^3^ CFU/mL. The plates were subsequently incubated at 35°C. The minimum inhibitory concentration (MIC) was defined as the lowest drug concentration at which complete growth inhibition was observed. Three replicates were conducted for each drug concentration.

### Isolating mutants using low dose of tunicamycin

To generate mutants, we inoculated approximately 2.5 × 10^3^ cells/mL of the H99 strain into 1.5 mL of YPD broth containing a low concentration of 0.06 μg/mL of TUN. We performed three biological replicates. After 72 h of incubation with shaking, the cultures were washed and diluted with distilled water. We then spread approximately 300 cells onto YPD plates and incubated them at 30°C for 72 h. From each culture, we randomly selected 120 colonies and tested them for resistance to TUN using a spot assay.

### Induction of mutations with high-dose tunicamycin

To generate mutants, we suspended cells in distilled water and adjusted the concentration to 1 × 10^7^ cells/mL. We then spread 100 μL of this suspension onto YPD plates supplemented with a high concentration of 0.25 μg/mL TUN. The plates were incubated at 30°C for 5 days, allowing for the selection of resistant mutants. We randomly selected 30 mutants for further analysis and characterization. These 30 mutants were maintained in a −80°C freezer.

### Assay for genome instability

To investigate the genome instability of the aneuploid strain with disomies of Chromosomes 4 and 6, we performed the following steps: First, the strain with Chrs (4,6) x2 was thawed from the-80°C freezer and streaked onto YPD-agar plates, which were then incubated at 30°C for 72 h. From the resulting colonies, we randomly selected one small colony and suspended it in distilled water. We then diluted the cells in distilled water and spread approximately 200 cells onto a YPD plate, which was incubated at 30°C for 72 h. After incubation, we observed small (S), medium (M), and large (L) colonies. We randomly selected four colonies from each size category for further analysis.

### Next-generation sequencing

DNA extraction, library construction, and sequencing were performed using previously described methods (Yang et al., 2021d). The data was then visualized using Ymap (Abbey et al., 2014). The raw fastq files were uploaded to Ymap (version 1.0)^1^ and plotted as a function of chromosome position using the *C. neoformans* H99 reference genome (NCBI RefSeq assembly no. GCF_000149245.1).

### RNA-seq

Comparative analysis of strains: To investigate the transcriptome profiles of the strains, we performed RNA-seq as described previously with minor modifications (Ke et al., 2024). We first streaked the strains onto YPD plates from the −80°C freezer and incubated them at 30°C for 72 h. We then selected colonies with similar sizes, suspended them in a solution with an optical density (OD) of 0.1, and grew them in a shaker at 30°C until they reached an OD of 1.0. The cells were then collected by centrifugation and frozen in liquid nitrogen.

Treatment of H99 with TUN: We grew H99 in YPD broth in a shaker at 30°C from an OD of 0.1 to 1.0, and then divided the culture into two batches. One batch was treated with TUN at a final concentration of 0.25 μg/mL, while the other batch was treated with an equal volume of DMSO. Three hours later, the cells were collected by centrifugation and frozen in liquid nitrogen.

Total RNA extraction, purification, library construction, and sequencing were performed as described previously (Ke et al., 2024). We obtained three biological replicates for each strain and used DESeq2 (Love et al., 2014) to analyze the differential gene expression profiles. We considered genes with a False Discovery Rate (FDR)-adjusted *p* value <0.05 and expression fold changes of more than 1.5 or less than −1.5 as differentially expressed.

### Statistical analysis

Significance analysis of differences between growth curves was performed using Tukey HSD (Honestly Significant Difference) test.

## Results

### Tunicamycin impact on *C. neoformans* H99 growth

**Figure 1:**
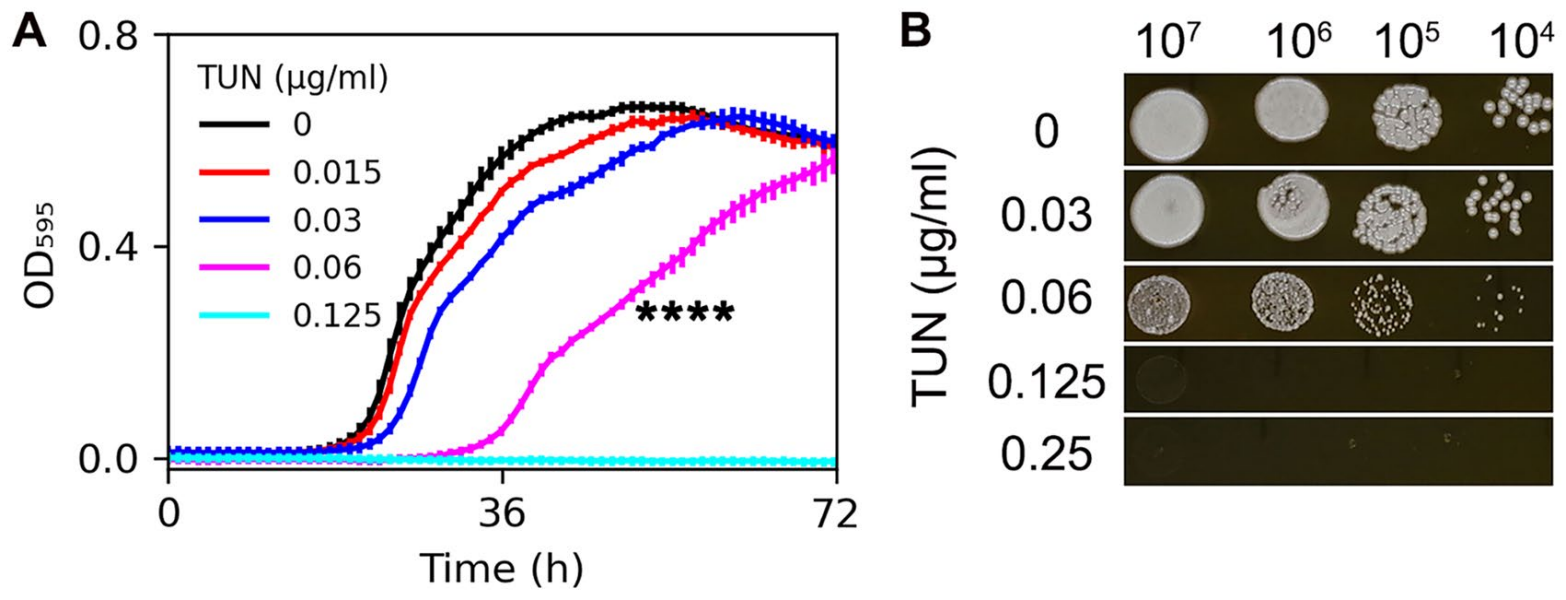
Assessment of H99 susceptibility to tunicamycin. Susceptibility was evaluated in both broth and solid medium using twofold increased concentrations of TUN. (A) Cells were cultured in a 96-well plate. Optical density at 595 nm (OD_595_) was monitored every hour for 72 h at 30°C using a Tecan plate reader. Data are presented as the mean ± SD of three biological replicates. (B) 3 μL of 10-fold serial dilutions of cell suspensions were spotted on YPD agar plates supplemented with TUN. The plates were then incubated at 30°C for 3 days and subsequently photographed.

The sensitivity of the *C. neoformans* lab strain H99 to TUN was evaluated. In YPD broth, the minimum inhibitory concentration (MIC) of TUN was found to be 0.125 μg/mL. Additionally, TUN at a concentration of 0.06 μg/mL significantly inhibited the growth of H99 (*p* < 0.001, Tukey test), while TUN at a concentration of 0.03 μg/mL did not significantly inhibit growth (*p* > 0.05, Tukey test) (Figure 1A).

On YPD agar plates, H99 was able to grow in the presence of TUN at a concentration of 0.06 μg/mL, although the resulting colonies were smaller than those on the control plate. TUN at concentrations of 0.125 μg/mL and 0.25 μg/mL completely inhibited the growth of H99 (Figure 1B).

Based on these results, TUN at a concentration of 0.06 μg/mL in YPD broth was found to be sub-inhibitory to H99, while TUN at a concentration of 0.125 μg/mL on YPD agar (and in YPD broth) was inhibitory to H99.

### Selective pressure of sub-MIC tunicamycin leads to emergence of antifungal resistant mutants

**Figure 2:**
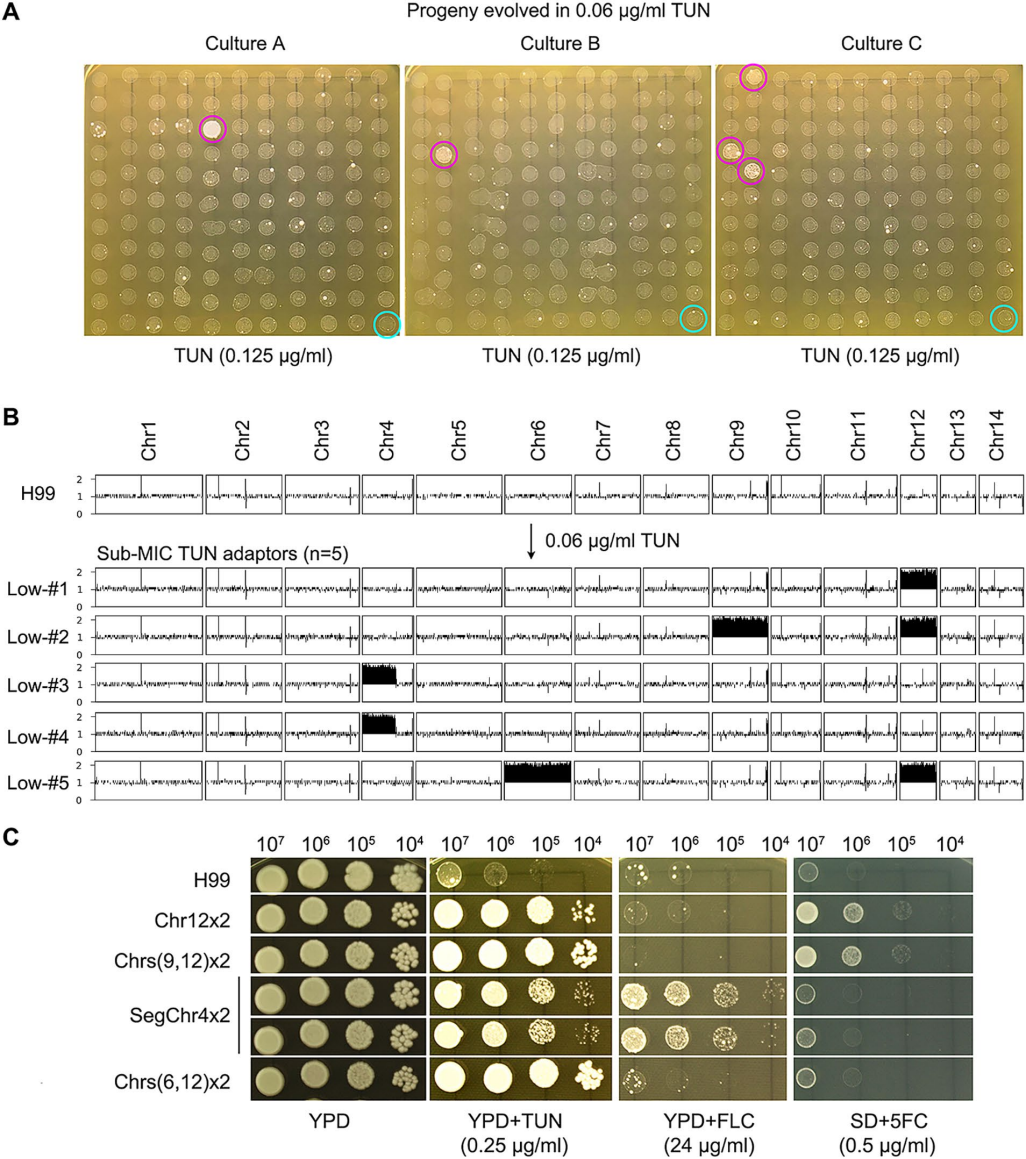
Selection of aneuploid mutants with antifungal resistance by low concentrations of tunicamycin. (A) The wild-type strain H99 was cultured in YPD broth supplemented with sub-minimal inhibitory concentration (sub-MIC) of TUN. Three biological replicates were conducted, and 120 colonies from each culture were randomly selected for testing their resistance to TUN (magenta circles) compared to the wild type (cyan circles). The five colonies displaying resistance were subjected to sequencing. (B) The karyotypes of the wild type and the five resistant colonies were visualized using Ymap. The y-axis represents copy number, while the x-axis indicates the location of the sequence reads on the chromosome. (C) The five mutants were subjected to spot assay to evaluate their resistance to TUN, fluconazole (FLC), and 5-flucytosine (5FC). Testing for 5FC was conducted on SD medium, while YPD medium was used for other tests.

We explored whether sub-MIC concentrations of TUN could select for adaptive mutants in the H99 strain. We cultured the strain in YPD broth supplemented with 0.06 μg/mL TUN, starting with a cell density of approximately 2.5 × 10^3^ cells/mL. After 72 h, we randomly selected 120 colonies from each culture and tested them for TUN susceptibility, with three biological replicates. Among these colonies, we identified five mutants that outperformed the parent strain (Figure 2A). The broth microdilution assay revealed that the parent strain H99 exhibited a MIC of 0.125 μg/mL for TUN, whereas the five mutants displayed MICs ranging from 0.5 to 1 μg/mL. Consequently, the five mutants demonstrated greater resistance to TUN compared to the parent strain.

Sequencing analysis of five selected mutants revealed diverse aneuploid karyotypes: Low-#1 had disomy of Chr12 (Chr12x2), Low-#2 had disomies of Chr9 and Chr12 (Chrs (9,12) x2), Low-#5 had disomies of Chr6 and Chr12 (Chrs (6,12) x2), and Low-#3 and Low-#4 had segmental disomy of the left arm of Chr4 (SegChr4x2) (Figure 2B). Thus, exposure to sub-inhibitory levels of TUN led to the selection of aneuploid TUN-resistant mutants with diverse karyotypes.

Variation analysis revealed few genetic mutations in the mutants (Supplementary Table S1). Low-#1 had a missense mutation in CNAG_06222, encoding the large subunit ribosomal protein L32e, while Low-#3 had a missense mutation in CNAG_00807, whose function is unknown.

We further examined whether the mutants displayed cross-resistance to antifungal drugs. Spot assays demonstrated that mutants with SegChr4x2 were more resistant to FLC than H99, while mutants with Chr12x2 and Chrs (9,12) x2 were more resistant to 5FC than H99 (Figure 2C). The broth microdilution assay indicated that the parent strain H99 had a MIC of 32 μg/mL for FLC, while the two mutants containing SegChr4x2 exhibited MICs greater than 128 μg/mL. For 5FC, H99 displayed an MIC of 0.5 μg/mL, whereas the mutants with Chr12x2 and Chrs (9,12) x2 had MICs of 4 μg/mL.

Thus, depending on the aneuploid chromosome, certain mutants exhibited cross-resistance to antifungals.

### Exposure to high concentrations of tunicamycin selects for diverse aneuploid mutants

**Figure 3:**
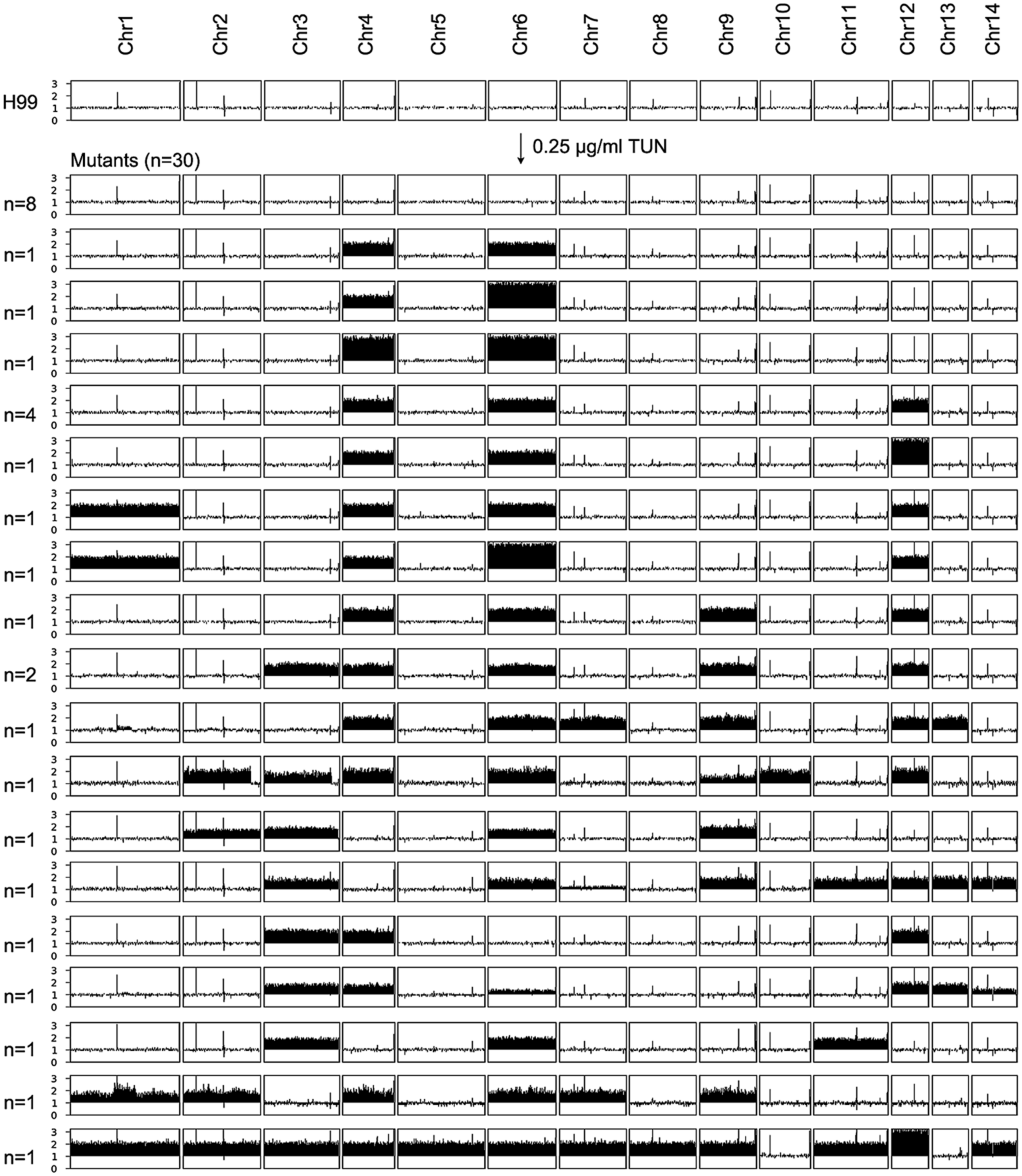
Karyotypes of mutants selected by high concentrations of tunicamycin. The wild-type strain H99 was exposed to supra-minimal inhibitory concentration (supra-MIC) of TUN. Thirty mutants were randomly selected for sequencing. The karyotypes of these mutants were visualized using Ymap, and the number of mutants bearing each karyotype is indicated in the figure.

We plated approximately 1 million H99 cells on YPD plates containing 0.125 μg/mL and 0.25 μg/mL TUN, respectively. After 5 days, we observed lawn growth on the plate with 0.125 μg/mL TUN, while 92 colonies grew on the plate with 0.25 μg/mL TUN. We randomly selected 30 mutants from the latter plate for further analysis. Whole-genome sequencing revealed that 8 mutants had a normal euploid karyotype, while 22 mutants displayed diverse aneuploid karyotypes, consisting of 18 distinct patterns. Notably, 17 out of 22 aneuploids exhibited disomy or trisomy of Chromosomes 4 and 6, either alone or in combination with other chromosomes. Five aneuploids had either Chr4x2 or Chr6x2, but not both, along with disomy of 2–6 other chromosomes (Figure 3). This suggests that amplifications of Chr4 and Chr6, alone or in combination with other chromosomes, were the primary genomic changes among mutants selected under high TUN concentration.

We identified several genetic mutations in the mutants (Supplementary Table S1). For example, mutants high-#8 and high-#12 had the same frameshift mutation in gene CNAG_06130, while mutants high-#15, high-#17, and high-#26 had missense mutations in CNAG_05936, CNAG_01727, and CNAG_05396, respectively. Mutant high-#20 had a missense mutation in CNAG_04310 and a stop-gained mutation in CNAG_06382, while mutant high-#29 had missense mutations in CNAG_03862 and CNAG_03059. However, the functions of proteins encoded by CNAG_06130, CNAG_04310, CNAG_03862, and CNAG_03059 remain unknown.

### Some supra-MIC mutants are cross-resistant to fluconazole and/or 5-flucytosine

**Figure 4:**
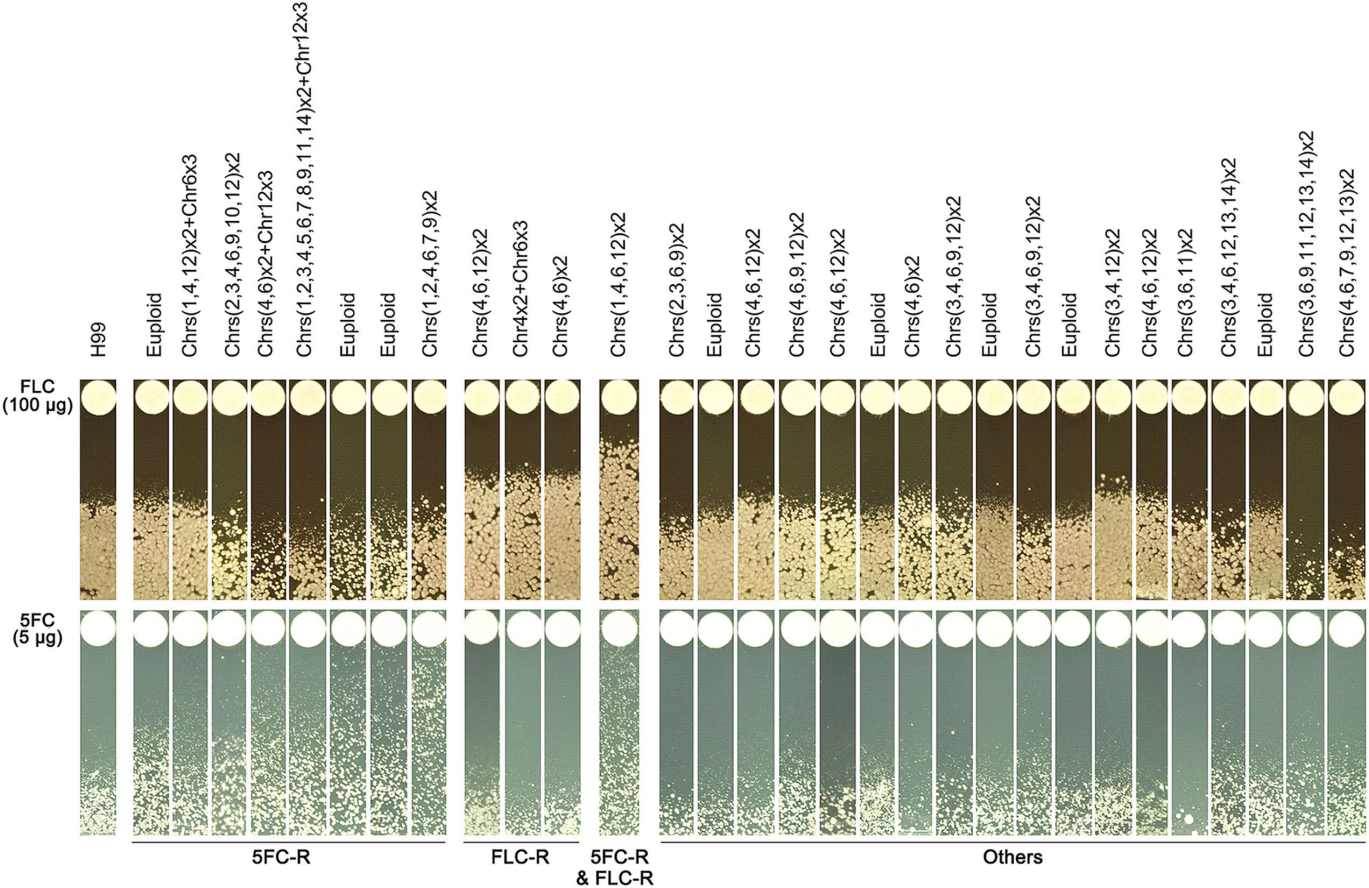
Profile of antifungal resistance in mutants induced by supra-MIC tunicamycin. Thirty mutants, induced by supra-minimal inhibitory concentration (supra-MIC) of TUN, were tested using disk diffusion assays. The top panel shows disks containing 200 μg of fluconazole (FLC), while the bottom panel shows disks containing 5 μg of 5-flucytosine (5FC). The diameter of the inhibition zone is inversely proportional to the level of drug resistance. The plates were incubated at 30°C for 3 days and then photographed. YPD plates were used for FLC testing, and SD plates were used for 5FC testing.

We found that some mutants that grew in the presence of high concentrations of TUN also showed resistance to other antifungal drugs, including FLC and 5FC. To further investigate this, we tested the antifungal resistance of all these mutants using disk diffusion assays. Based on their resistance profiles, we categorized the mutants into four distinct groups (Figure 4).

One group of mutants was resistant to 5FC only and consisted of 8 mutants, which had MICs for 5FC ranging from 1 to 8 μg/mL. Interestingly, three of these mutants had a normal euploid karyotype, while the remaining five had amplifications of at least three chromosomes.

Another group of mutants was resistant to FLC only and consisted of three mutants, each having an MIC of 64 μg/mL for FLC. Each of these mutants had distinct chromosomal changes, including disomy of Chromosomes 4 and 6, disomy of Chromosome 4 and trisomy of Chromosome 6, and disomy of Chromosomes 4, 6, and 12.

A third group of mutants showed cross-resistance to both 5FC and FLC, and consisted of only one mutant. The MICs for FLC and 5FC were 128 μg/mL and 8 μg/mL, respectively. This mutant had a complex karyotype, with disomy of Chromosomes 1, 4, 6, and 12.

The final group of mutants did not show obvious resistance to either 5FC or FLC, and consisted of 18 mutants. However, some of these mutants were hypersensitive to FLC, while others were hypersensitive to 5FC.

### Aneuploidy is unstable

**Figure 5:**
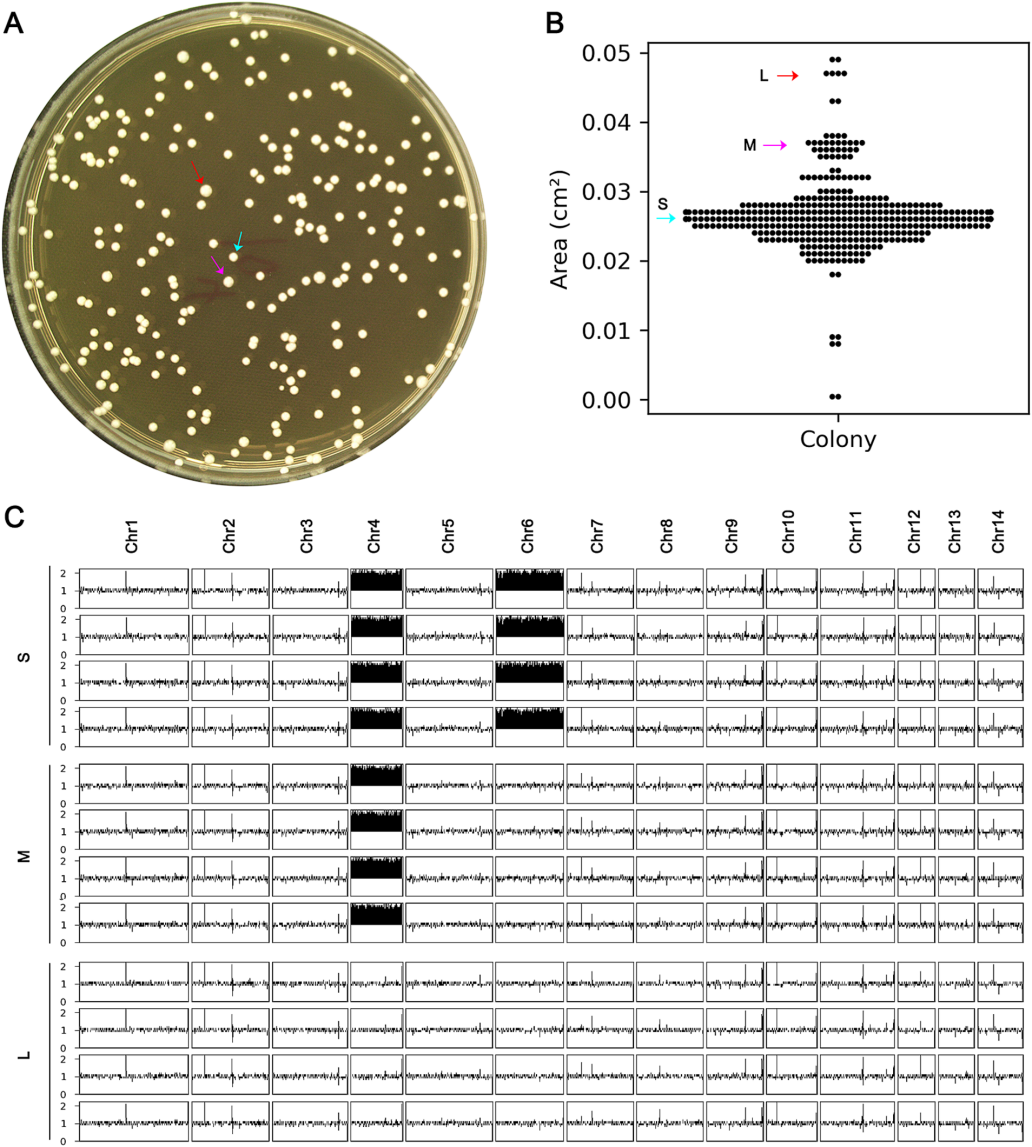
Instability of complex aneuploidy. (A) A mutant with disomies of Chr4 and Chr6 was spread on YPD plate. (B) After 48 h of growth, the areas of the colonies were measured. (C) Colonies were classified as small (S), medium (M), or large (L), indicated by cyan, magenta, and red arrows, respectively. Four colonies of each category were randomly selected for sequencing, and the karyotypes were visualized using Ymap.

A mutant with a chromosomal abnormality, Chrs (4,6) x2, was analyzed for its physical characteristics and genetic stability. To do this, around 400 cells of the mutant were grown on a special agar plate (Figure 5A). After two days, differences in the size of the resulting colonies were observed (Figure 5A). The area of each colony was then measured (Figure 5B). Colonies that were smaller than 0.03 cm^2^ were classified as small, those between 0.035 cm^2^ and 0.04 cm^2^ as medium, and those larger than 0.045 cm^2^ as large (Figure 5A,B). Four colonies from each size group were randomly selected for further genetic analysis. The results showed that all four small colonies still had the Chrs (4,6) x2 abnormality, while the four medium colonies had a different abnormality, Chr4x2, and the four large colonies had a normal chromosome count (Figure 5C). This suggests that the Chrs (4,6) x2 mutant is unstable and may tend to lose the extra copy of chromosome 6 or all extra chromosomes.

Randomly selected S, M, and L colonies, one from each type, were compared to the H99 strain for resistance to TUN. The spot assay indicated that both S and M colonies exhibited resistance, while the L colony did not (Supplementary Figure S1). Thus, the presence of an additional copy of Chr4 alone was sufficient to confer resistance to TUN, and the loss of all extra copies of aneuploid chromosomes resulted in a loss of TUN resistance.

### Aneuploidy causes cross-resistance to tunicamycin and fluconazole via regulating expression of multiple genes across the genome

**Figure 6:**
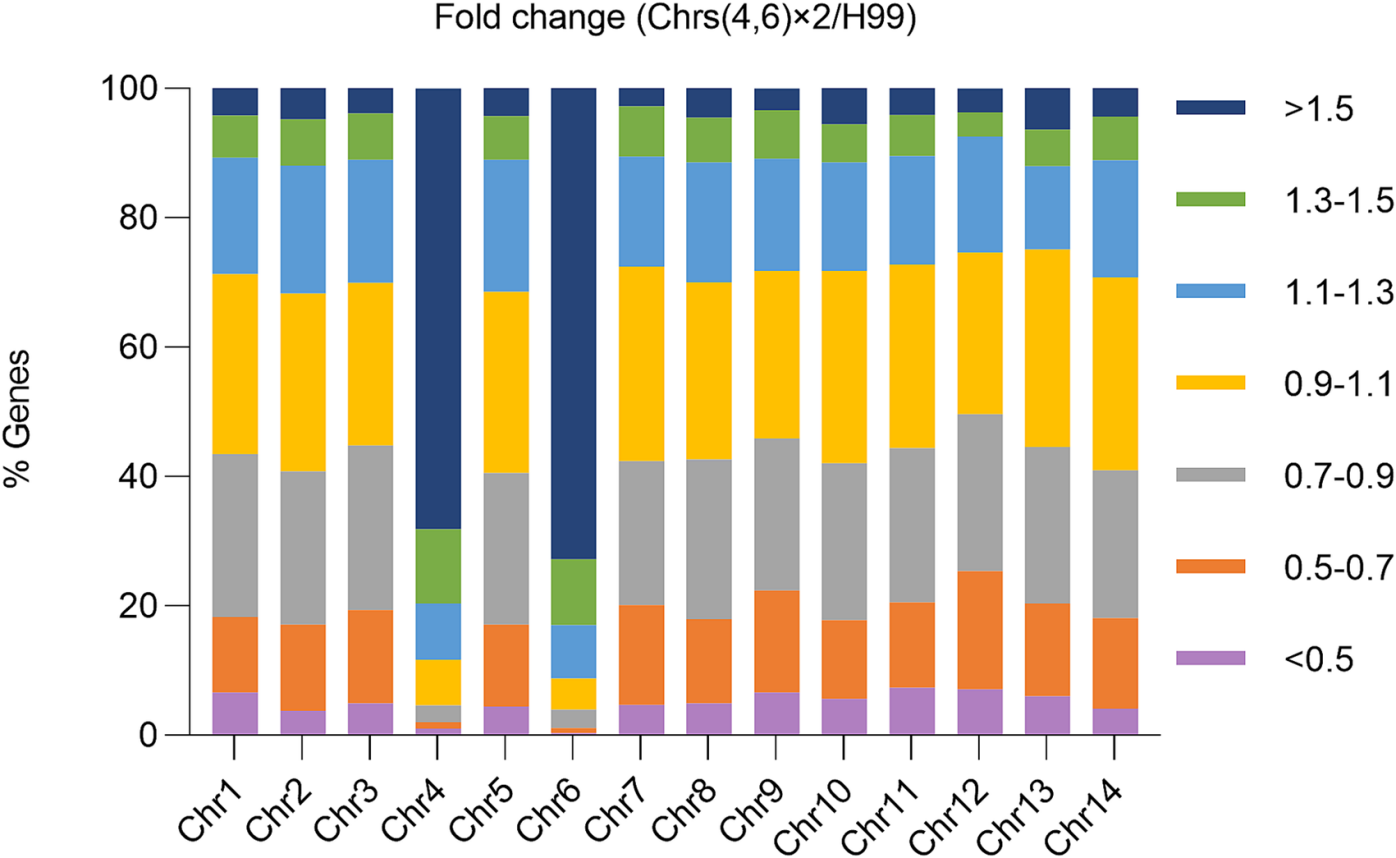
Impact of aneuploidy on transcription of genes across the genome. The transcriptome of a mutant with Chrs (4,6) x2 was compared to the haploid wild type H99. Relative expression was determined based on the expression ratio of a target gene in the aneuploid strain versus in H99. The graph displays the proportions of genes on each chromosome falling within specified relative expression ratio ranges.

Our study reveals that disomy of Chrs (4,6), either alone or in combination with disomy of other chromosomes, was the primary genomic alteration among the mutants. Additionally, Chrs (4,6) x2 conferred resistance not only to TUN but also to FLC. To elucidate why Chrs (4,6) x2 led to cross-resistance to both drugs, we compared the transcriptome of one Chrs (4,6) x2 mutant to that of the wild type (Figure 6).

Transcription of 392 genes was detected on Chr4, with 312 genes showing ratios higher than 1.3. Similarly, transcription of 533 genes was detected on Chr6, with 442 genes having ratios higher than 1.3. Consequently, 79.6 and 82.9% of transcribed genes on Chr4 and Chr6, respectively, were up-regulated at least 1.3 times in the Chrs (4,6) x2 strain compared to H99. In contrast, on euploid chromosomes, only 7.5–12.1% of genes were up-regulated to this extent.

Overall, 910 genes were up-regulated across the genome, with 267 and 388 genes on Chr4 and Chr6, respectively. Conversely, 990 genes were down-regulated, with only 8 and 4 on Chr4 and Chr6, respectively. Thus, 72.0% (655 out of 910) of the up-regulated and 1.2% (12 out of 990) of the down-regulated genes were located on aneuploid chromosomes. Consequently, disomy of Chr4 and Chr6 generally led to increased expression of genes on the aneuploid chromosomes and altered expression of genes on euploid chromosomes.

Gene ontology (GO) analysis revealed that 21 genes involved in response to TUN (GOID: 1904576) were significantly enriched among the up-regulated genes, with 14 of them located on Chr4 or Chr6 (Supplementary Table S2). Notably, among the genes encoding efflux pumps, *AFR1*/CNAG_00730, *MDR1*/CNAG_00796, and *PDR16*/CNAG_04984 were up-regulated, with *AFR1* and *MDR1* on Chr1 and *PDR16* on Chr4. Among the *ERG* genes, only *ERG19*/CNAG_05125 and *ERG20*/CNAG_02084 were up-regulated, with *ERG19* on Chr4 and *ERG20* on Chr6. Conversely, *ERG4*/CNAG_02830 was down-regulated (Supplementary Table S2). However, other *ERG* genes, such as *ERG11*/CNAG_00040, *ERG3*/CNAG_00519, or *ERG6*/CNAG_03819, showed no differential expression in Chrs (4,6) x2 compared to H99. Thus, we propose that Chrs (4,6) x2 confers resistance to TUN and FLC by simultaneously up-regulating multiple genes on both euploid and aneuploid chromosomes.

### Exposure to tunicamycin leads to cross-resistance to fluconazole in a clinical isolate of *C. neoformans*

**Figure 7:**
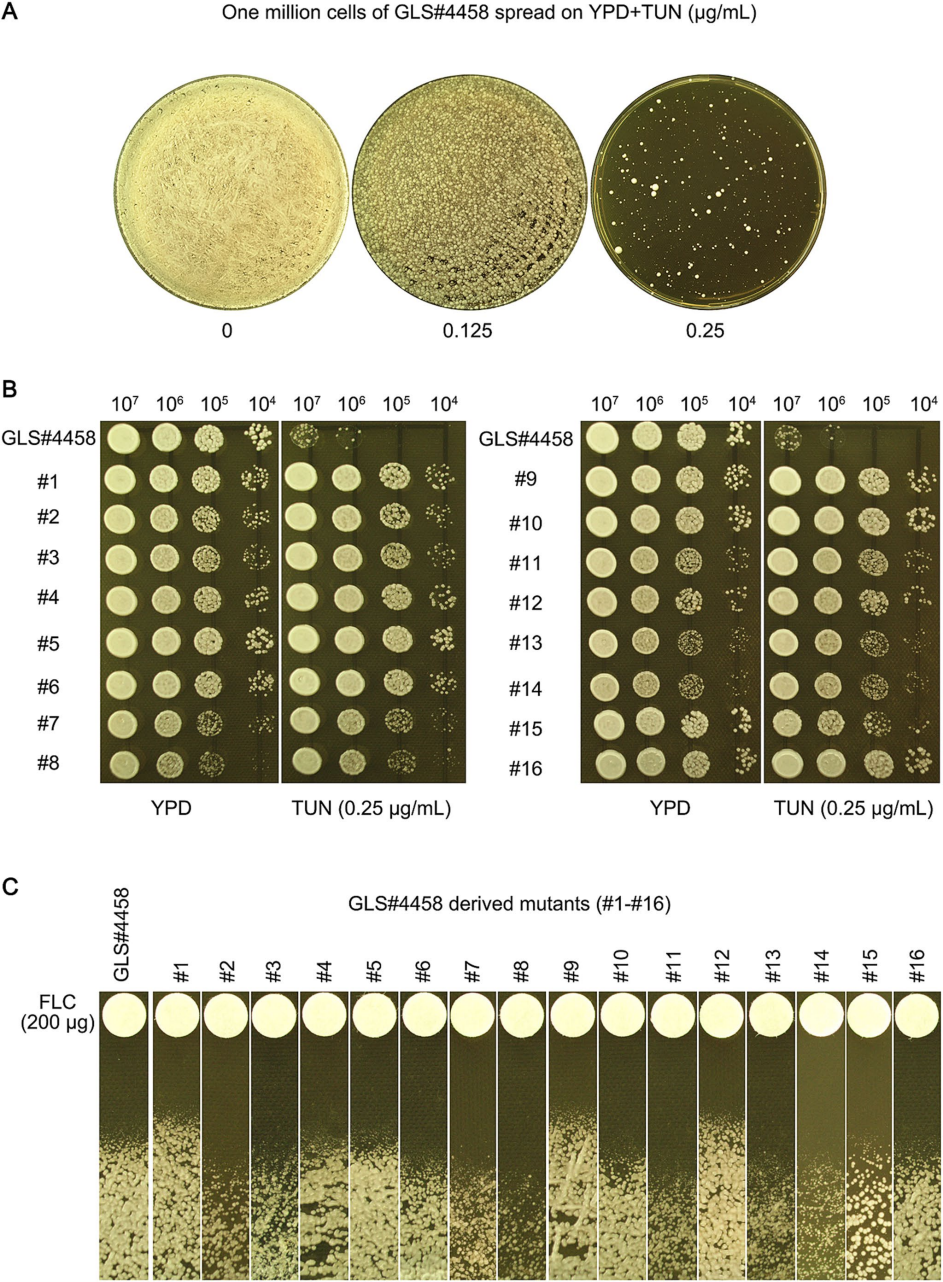
Tunicamycin induces fluconazole resistance in clinical isolate of *C. neoformans*. (A) GLS#4458 is a clinical isolate of *C. neoformans*. The cells of GLS#4458 were suspended in distilled water and adjusted to a concentration of 1.0 × 10^7^ cells/mL. A volume of 100 μL of the cell suspension was spread onto YPD-agar medium supplemented with TUN, with drug concentrations indicated in the figure. The plates were incubated at 30°C for 5 days before being photographed. Sixteen colonies (#1–#16) were randomly selected from the plate containing 0.25 μg/mL for further analysis. (B) A spot assay was conducted to compare the resistance to TUN between the 16 mutants and the wild type. For each strain, 3 μL of 10-fold serial dilutions were spotted on YPD-agar plates with 0.25 μg/mL TUN or without TUN (as a control). The plates were then incubated at 30°C for 3 days and subsequently photographed. (C) In the disk diffusion assay, disks containing 200 μg FLC were used. The plates were incubated at 30°C for 3 days before being photographed.

We investigated whether TUN also induced antifungal resistance in clinical isolates. Approximately 1 million cells of the clinical isolate GLS#4458 were spread on YPD-agar plates supplemented with TUN. After 5 days, the plate with 0.125 μg/mL TUN showed lawn growth, while the plate with 0.25 μg/mL TUN displayed a few 100 visible colonies (Figure 7A). Sixteen mutants (#1–#16) were randomly selected. Spot assays revealed that all of them were able to grow in the presence of 0.25 μg/mL TUN, whereas growth of the wild-type GLS#4458 was completely inhibited (Figure 7B), indicating that all the mutants developed resistance to TUN. Disk assays were conducted using disks containing 200 μg FLC, and three mutants, #1, #9, and #12, exhibited smaller inhibition zones compared to the parent strain (Figure 7C), suggesting that these three mutants acquired resistance to FLC.

## Discussion

ER is a fundamental organelle found in eukaryotic cells. Traditionally, studies investigating ER stress induced by chemical agents have mainly focused on elucidating the associated signaling pathways. However, recent research has uncovered a connection between ER stress and the formation of aneuploidy in yeast species, with subsequent implications for the fortuitous development of antifungal resistance (Beaupere et al., 2018; Yang et al., 2021b). To our knowledge, our study represents the first direct link between ER stress and the emergence of antifungal resistance, including cross-resistance, in *C. neoformans*. It’s important to note that while aneuploidy itself generally does not confer resistance to various stresses, including antifungal drugs, it’s the specific aneuploidy of particular chromosome (s) that can alter susceptibility to specific stressors.

The mechanisms underlying TUN-induced ER stress and its subsequent role in aneuploidy formation in *C. neoformans* remain elusive. Aneuploidy provides a fitness advantage under stress conditions by directly regulating genes on the aneuploid chromosome or indirectly impacting genes on euploid chromosomes (Pavelka et al., 2010). In species like *S. cerevisiae* and *C. albicans*, TUN-induced amplification of specific chromosomes is attributed to the presence of particular genes, such as *ALG7* (Beaupere et al., 2018; Yang et al., 2021b). However, in the genome of *C. neoformans*, *ALG7*/CNAG_06901 is located on Chr3, and only a minority of mutants exhibit amplification of Chr3. Instead, amplification of Chr4 and Chr6 emerges as the predominant genomic alteration among the mutants. Gene ontology (GO) analysis has identified at least 14 genes on Chr4 and Chr6 associated with resistance to TUN (refer to Supplementary Table S2). Future investigations will focus on determining whether amplification of these genes in the wild type is sufficient to confer resistance to TUN.

In the Chrs (4,6) x2 strain, which exhibited resistance to FLC, several genes encoding efflux pumps, such as *AFR1* and *MDR1* on Chr1, as well as *PDR16* on Chr4, were up-regulated compared to the wild type. However, most of the *ERG* genes, including *ERG11*, showed no differential expression. We propose that Chrs (4,6) x2 induces cross-resistance to TUN and FLC by concurrently up-regulating multiple genes on both aneuploid and euploid chromosomes.

The TUN-selected mutants exhibit diverse genomic alterations, including increases in copy number across various chromosomes and to varying extents, while showing few genetic mutations, suggesting that TUN primarily induces genome instability rather than hypermutation in *C. neoformans*. ER stress and genome damage are interconnected, as ER stress can induce genome instability by impeding the repair of double-strand breaks (DSBs) (Yamamori et al., 2013). DSBs are particularly detrimental to genome integrity (Blackford and Jackson, 2017), and their unrepaired status contributes to genome instability, leading to chromosome missegregation and aneuploidy formation (Srivastava and Raghavan, 2015; Blackford and Jackson, 2017).

Our study illustrates that brief exposure to mild ER stress is adequate to trigger aneuploidy in *C. neoformans*, mirroring the previous findings in *C. albicans* (Yang et al., 2021b). Additionally, besides ER stress, short-term exposure to sub-inhibitory concentrations of FLC also prompts aneuploidy in *C. neoformans* (Yang et al., 2021a) and *C. albicans* (Sun et al., 2023b; Todd et al., 2023; Yang et al., 2023). Thus, we propose that the genome plasticity of fungal pathogens can be readily and swiftly induced by mild stresses.

Our study underscores the genome plasticity of *C. neoformans* as a swift and reversible mechanism for adapting to TUN-induced ER stress, offering fresh insights into the mechanism underlying the emergence of antifungal resistance.

## Data Availability

The datasets presented in this study can be found in online repositories. The names of the repository/repositories and accession number(s) can be found below: https://www.ebi.ac.uk/arrayexpress/, E-MTAB-12268, E-MTAB-12276, EMTAB- 12286 and E-MTAB-12275.

## Footnotes

^1^http://lovelace.cs.umn.edu/Ymap/
