# Supplementary figures and images for: Unveiling genome plasticity as a mechanism of non-antifungal-induced antifungal resistance in Cryptococcus neoformans
Source: Front Microbiol. 2024 Nov 5;15:1470454. doi: 10.3389/fmicb.2024.1470454 (PMC11573520; doi:10.3389/fmicb.2024.1470454)

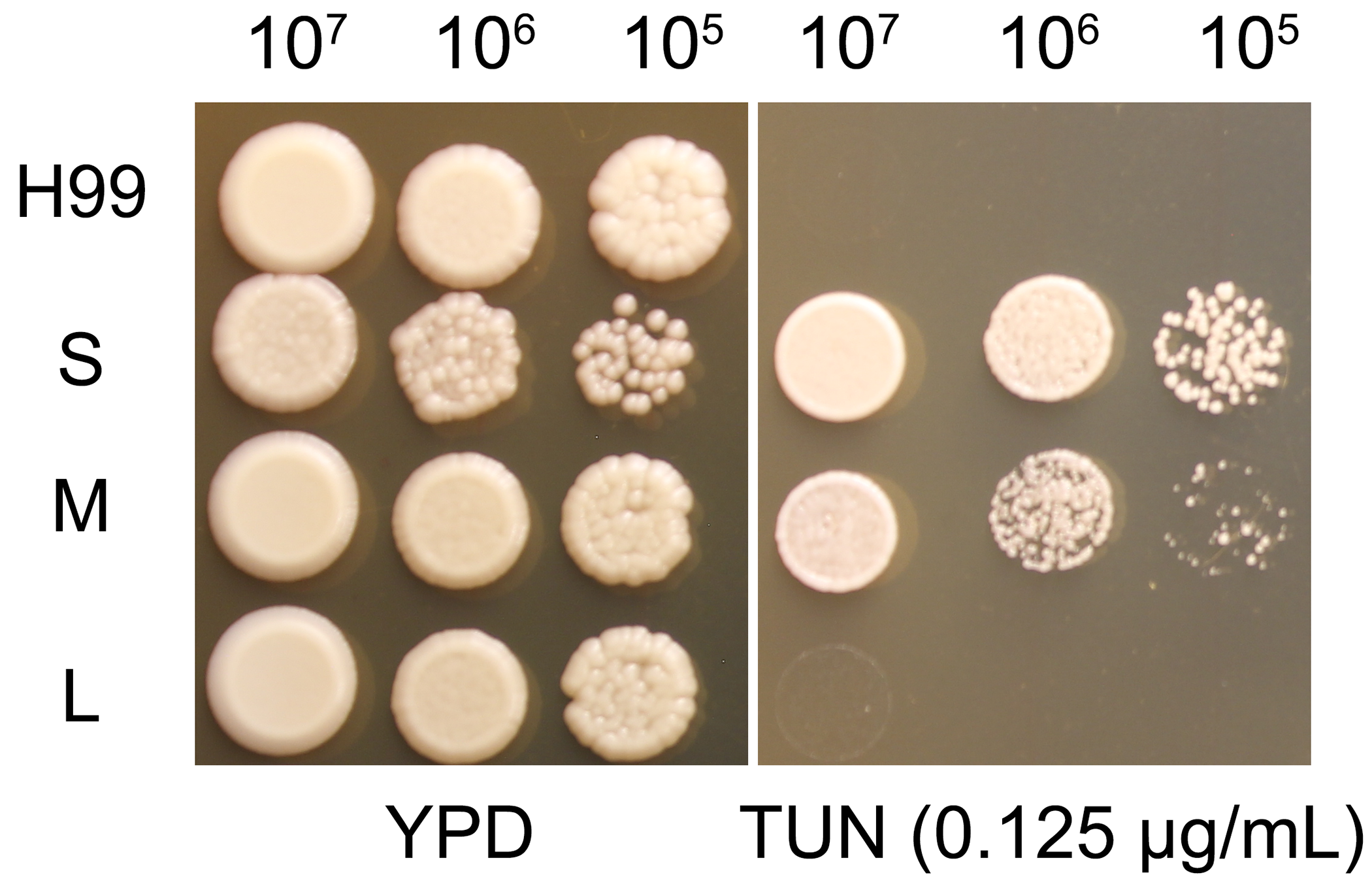

Supplement: Supplementary file 1 [file Image_1.TIF]
