# Supplementary material for: Unveiling genome plasticity as a mechanism of non-antifungal-induced antifungal resistance in Cryptococcus neoformans
Source: Front Microbiol. 2024 Nov 5;15:1470454. doi: 10.3389/fmicb.2024.1470454 (PMC11573520; doi:10.3389/fmicb.2024.1470454)
Supplement: Supplementary file 2 [file Table_1.DOCX]

**Supplemental materials**

**Table S1. Mutations identified in tunicamycin-induced mutants**

| Strain | Gene | Position | Ref | Alt | Type | Effect |
| --- | --- | --- | --- | --- | --- | --- |
| Low-#1 | CNAG_06222 | 669406 | C | T | SNP | Missense |
| Low-#3 | CNAG_00807 | 2134455 | C | T | SNP | Missense |
| High-#8 | CNAG_06130 | 385350 | T | TA | INDEL | Frameshift |
| High-#12 | CNAG_06130 | 385350 | T | TA | INDEL | Frameshift |
| High-#15 | CNAG_05936 | 1256932 | C | G | SNP | Missense |
| High-#17 | CNAG_01727 | 730685 | G | C | SNP | Missense |
| High-#20 | CNAG_04310 | 533289 | T | C | SNP | Missense |
| High-#20 | CNAG_06382 | 346595 | C | T | SNP | Stop gained |
| High-#26 | CNAG_05396 | 203768 | T | G | SNP | Missense |
| High-#29 | CNAG_03862 | 1036084 | G | A | SNP | Missense |
| High-#29 | CNAG_03059 | 94948 | T | A | SNP | Missense |

**Table S2. Relative expression of genes involved in resistance to tunicamycin and fluconzole**

| **Gene ID** | **Gene** | **Chromosome** | **Ratio Chrs(4,6)x2/H99** |
| --- | --- | --- | --- |
| Genes involved in response to tunicamycin | | | |
| *CNAG_00559* | *BZP3* | Chr1 | 1.98 |
| *CNAG_03670* | *IRE1* | Chr2 | 1.56 |
| *CNAG_03963* | *OCA1* | Chr2 | 1.62 |
| *CNAG_02820* | *PKH201* | Chr3 | 1.53 |
| *CNAG_05019* | *FZC21* | Chr4 | 1.98 |
| *CNAG_05063* | *SSK2* | Chr4 | 2.08 |
| *CNAG_05153* | *GAT5* | Chr4 | 1.59 |
| *CNAG_05155* | *PTP2* | Chr4 | 1.83 |
| *CNAG_05159* |  | Chr4 | 2.35 |
| *CNAG_05170* | *P1P2* | Chr4 | 1.54 |
| *CNAG_05176* | *HOB3* | Chr4 | 1.84 |
| *CNAG_05222* | *NRG1* | Chr4 | 1.68 |
| *CNAG_05255* | *FZC2* | Chr4 | 1.65 |
| *CNAG_02109* | *INP5201* | Chr6 | 2.49 |
| *CNAG_02236* | *PPG1* | Chr6 | 2.1 |
| *CNAG_02357* | *MKK2* | Chr6 | 2.08 |
| *CNAG_02435* | *BWC2* | Chr6 | 1.6 |
| *CNAG_02470* | *PPP2* | Chr6 | 1.82 |
| *CNAG_02490* | *MRE11* | Chr6 | 1.73 |
| *CNAG_04630* | *YAP2* | Chr10 | 2.1 |
| *CNAG_04678* | *YPK1* | Chr10 | 1.59 |
| Genes involved in biosynthesis of ergosterol | | | |
| *CNAG_00040* | *ERG11* | Chr1 | 1.03 |
| *CNAG_00117* | *ERG24* | Chr1 | 0.77 |
| *CNAG_00854* | *ERG2* | Chr1 | 0.84 |
| *CNAG_00519* | *ERG3* | Chr1 | 0.73 |
| *CNAG_03819* | *ERG6* | Chr2 | 0.91 |
| *CNAG_02830* | *ERG4* | Chr3 | 0.62 |
| *CNAG_07510* | *ERG9* | Chr3 | 1.17 |
| *CNAG_02918* | *ERG10* | Chr3 | 1.02 |
| *CNAG_02896* | *ERG130* | Chr3 | 1 |
| *CNAG_05125* | *ERG19* | Chr4 | 1.78 |
| *CNAG_06829* | *ERG1* | Chr5 | 1.14 |
| *CNAG_01129* | *ERG7* | Chr5 | 1.27 |
| *CNAG_07437* | *ERG27* | Chr5 | 1.3 |
| *CNAG_02084* | *ERG20* | Chr6 | 1.63 |
| *CNAG_06644* | *ERG5* | Chr7 | 0.87 |
| *CNAG_03311* | *ERG13* | Chr8 | 1.08 |
| *CNAG_04605* | *ERG26* | Chr10 | 1.11 |
| *CNAG_01737* | *ERG25* | Chr11 | 1.03 |
| *CNAG_06001* | *ERG8* | Chr12 | 0.75 |
| Genes involved in efflux | | | |
| *CNAG_00730* | *AFR1* | Chr1 | 1.75 |
| *CNAG_00796* | *MDR1* | Chr1 | 3.41 |
| *CNAG_04984* | *PDR16* | Chr4 | 2.73 |
| *CNAG_07799* |  | Chr4 | 3.46 |
